# Leveraging a graft collection to develop metabolome-based trait prediction for the selection of tomato rootstocks with enhanced salt tolerance

**DOI:** 10.1093/hr/uhac061

**Published:** 2022-03-14

**Authors:** Chao Song, Tania Acuña, Michal Adler-Agmon, Shimon Rachmilevitch, Simon Barak, Aaron Fait

**Affiliations:** 1 The Albert Katz International School for Desert Studies, The Jacob Blaustein Institutes for Desert Research, Ben-Gurion University of the Negev, Sede Boqer Campus, 8499000, Israel; 2 Albert Katz Department of Dryland Biotechnologies, French Associates Institute for Agriculture and Biotechnology of Drylands, Jacob Blaustein Institutes for Desert Research, Ben-Gurion University of the Negev, Sede Boqer Campus, 8499000, Israel; 3 R&D Southern Arava, Hevel Eilot, 8882000, Israel

## Abstract

Grafting has been demonstrated to significantly enhance the salt tolerance of crops. However, breeding efforts to develop enhanced graft combinations are hindered by knowledge-gaps as to how rootstocks mediate scion-response to salt stress. We grafted the scion of cultivated M82 onto rootstocks of 254 tomato accessions and explored the morphological and metabolic responses of grafts under saline conditions (EC = 20 dS m^−1^) as compared to self-grafted M82 (SG-M82). Correlation analysis and Least Absolute Shrinkage and Selection Operator were performed to address the association between morphological diversification and metabolic perturbation. We demonstrate that grafting the same variety onto different rootstocks resulted in scion phenotypic heterogeneity and emphasized the productivity efficiency of M82 irrespective of the rootstock. Spectrophotometric analysis to test lipid oxidation showed largest variability of malondialdehyde (MDA) equivalents across the population, while the least responsive trait was the ratio of fruit fresh weight to total fresh weight (FFW/TFW). Generally, grafts showed greater values for the traits measured than SG-M82, except for branch number and wild race-originated rootstocks; the latter were associated with smaller scion growth parameters. Highly responsive and correlated metabolites were identified across the graft collection including malate, citrate, and aspartate, and their variance was partly related to rootstock origin. A group of six metabolites that consistently characterized exceptional graft response was observed, consisting of sorbose, galactose, sucrose, fructose, *myo*-inositol, and proline. The correlation analysis and predictive modelling, integrating phenotype- and leaf metabolite data, suggest a potential predictive relation between a set of leaf metabolites and yield-related traits.

## Introduction

Salt stress is one of the most important abiotic stresses hampering plant growth and affecting crop production and affects about 20% of irrigated land worldwide [1]. Moderate salinity (EC: 4–8 dS m^−1^) can reduce average yields by 50–80% and subsequently result in a yield gap for all major glycophytic crops [2], thereby leading to unsustainable growth rates of agricultural demand. The deleterious effects of soil salinity on plant growth mainly result from osmotic stress, ionic toxicity, nutritional imbalance, and oxidative damage [3]. Plants have evolved different strategies for protection against salinity including synthesis of compatible osmolytes, ion compartmentation, enhancement of enzymatic or non-enzymatic antioxidant systems, and changes in hormone levels and hormone-mediated signalling [4–7].

Considering that soil salinity poses a significant threat to agriculture, improving salt tolerance of crops and identifying the biochemical and molecular basis of salt tolerance are high-priority goals of scientific research and agricultural practices [4, 8, 9]. However, the complex genetic and physiological-based response to salt stress leave important unresolved questions [2, 10, 11]. Among the strategies to counter the detrimental effects of soil salinity on crops, grafting has shown important results in several species [12–15].

Grafting establishes a vascular continuity in a natural or deliberate fusion of plant parts and results in the genetically composite organism functioning as a single plant [16]. Currently, grafting is used in a number of crop species such as cucumber, watermelon, citrus, and various Solanaceae [17–22]. Grafting can boost plant growth, control wilt caused by pathogens, reduce viral, fungal, and bacterial infection, strengthen tolerance to thermal or saline stress, and increase nutrient and mineral uptake to the shoot [23]. It has been demonstrated that rootstocks can induce scion tolerance to salinity by comprehensively improving shoot performance (e.g. dry matter accumulation, leaf area, leaf water potential, and stomatal conductivity) [24–27].

Grafting in tomato has been mostly investigated in small-scale experiments, indicating the morphological [28–30], physiological [31, 32], and metabolic alterations [33] in the scion mediated by rootstocks. Gerieneisen et al. [34] summarised 159 publications using grafted tomatoes and found that 35% (294 of 684) of the heterografted plants produced significantly higher yields than the corresponding controls. The cultivar cv. Maxifort was the most commonly tested rootstock among 202 rootstocks in 1023 experimental treatments, comprising different grafts, locations, and growing seasons. By grafting the scion onto different rootstocks, salt tolerance in scion could be altered and improved [35], leading to enhanced plant growth [36], fruit yield, and fruit quality [37]. Improvement in salt tolerance manifested either as plant growth [38] or physiological aspects [39] of grafted tomatoes was due to interaction of the scion with the rootstock [40, 41]. However, to the best of our knowledge, no comprehensive investigation has been conducted regarding the metabolic response in plant leaves under sub-optimal conditions mediated by rootstock biodiversity and how rootstock-mediated leaf metabolism is associated with plant yield traits.

Metabolomics-assisted breeding has been proposed to accelerate breeding processes [42]. The potential application of metabolic markers has been suggested by robust, significant correlations between metabolites and at least one whole-plant phenotypic trait in tomato [43]. In tomato seeds, seed germination was found to be negatively correlated to amino acids such as proline, methionine, leucine, and lysine [44]. The redundancy of metabolic markers makes it difficult to use them as individual features. A more robust approach will be the production of metabolic signatures, whereby a group of metabolic features is found that is predictive of a yield/quality related trait [45]. In another report, the predictive ability, calculated as the Pearson’s correlation coefficient between the observed and predicted value, can reach as high as 0.977 in predicting agronomic traits using metabolites [46]. Taken together, metabolic prediction of phenotypic traits has being a approach of great potential addressing the association between metabolites and polygenic traits [47, 48].

In this study, we explored the effect of a collection of 254 tomato rootstock accessions on the morphological and metabolic traits of cv. M82 plants under saline soil conditions. We then tested predictive models to link the metabolic alteration mediated by the rootstocks in the plant leaves with its yield-related traits.

## Results

### Tomato grafts onto different rootstocks exhibit a broad spectrum of phenotypes under saline conditions

**Figure 1:**
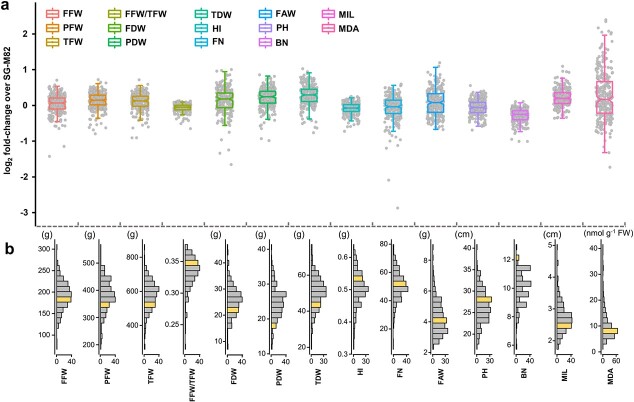
Variation of morphological traits and malondialdehyde (MDA) content across the grafted tomato population. **a** Each dot in the box plot represents the mean of the fold change of each tomato for the respective parameters over self-grafted M82 (SG-M82), followed by log_2_ transformation (n = 3–4). The line across each box shows the median of each dataset. The notch of each boxplot indicates the 95% confidence interval of dataset. **b** The histogram shows the data distribution of tomato populations of each parameter. The bin colored with gold indicates where the SG-M82 is located. FFW, fruit fresh weight; PFW, plant fresh weight; TFW, total fresh weight; FFW/TFW, the ratio of fruit fresh weight to total fresh weight. FDW, fruit dry weight; PDW, plant dry weight; TDW, total dry weight; HI, harvest index; FAW, fruit average weight; PH, plant height; BN, branch number; MIL, mean internode length; MDA, malondialdehyde.

The log_2_ FCs of each graft against SG-M82 were calculated to visualize the effect of rootstocks on scion performance under saline (NaCl) irrigation; thus, positive and negative values show the increases and decrease over control, respectively (Fig. 1). Heterogeneity in morphological traits and in the relative content of malondialdehyde (MDA) as an indicator of oxidative damage in tomato under salt stress [49, 50] was observed as a result of the rootstock-mediated response of M82 (Fig. 1a). To evaluate the extent of variation for each trait, a coefficient of variation (CV) was calculated as the ratio of standard deviation over the mean of each trait across the entire grafted population (Fig. S2). Hence, the higher the CV, the greater the variability of a given trait mediated by a rootstock. We observed that MDA content had the highest CV value (CV = 0.58), showing an FC range of 0.30 to 5.21 compared to SG-M82, whereas the FFW/TFW ratio and harvest index were the traits with the lowest CV (CVs = 0.07 and 0.11, respectively) across the entire population, presenting narrow FC range of 0.63 to 1.08 (Fig. 1a). These data suggest relative resilience of shoot and yield-related traits to grafting.

The diagram (Fig. 1b), in which the population is divided into 20 bins for each trait, shows the effects of rootstocks on morphological traits. Almost all grafts, 98.4% (250 of 254), generated fewer branches (BN) than SG-M82 (gold bin). When considering the overall performance of the plants, we classified the plants according to MDA content and morphological traits. As such, more than 200 grafts showed better performance (i.e. greater values for morphological traits and lower values for MDA content) than SG-M82, accumulated higher PDW (n = 215), TDW (n = 208), and longer MIL (n = 209), as indicated by the skew relative to SG-M82. Next, grafts were separated into 14 bins corresponding to 14 measured traits including MDA (Fig. S3). Fig. S3a shows the frequency of grafted lines that exhibited better performance than SG-M82 for each of the 14 traits. For instance, 19 grafts displayed comprehensive improvements in 12 out of 14 plant growth traits under saline conditions compared to SG-M82. None of the 254 grafts exhibited better performance than SG-M82 over all 13 or 14 traits. The 254 tomato accessions used as rootstocks for plant grafting were from five different origins, occupying different proportions in the grafted population (Fig. S3b). The proportions of rootstock origin in each bin were similar to that in the grafted population, except for the 17 wild species, which were mainly associated with smaller plant growth parameters, such as lower FFW and TFW (Fig. S4). This consistent pattern indicates that domestication led to relative homogeneity in supporting scion growth under saline conditions.

**Figure 2:**
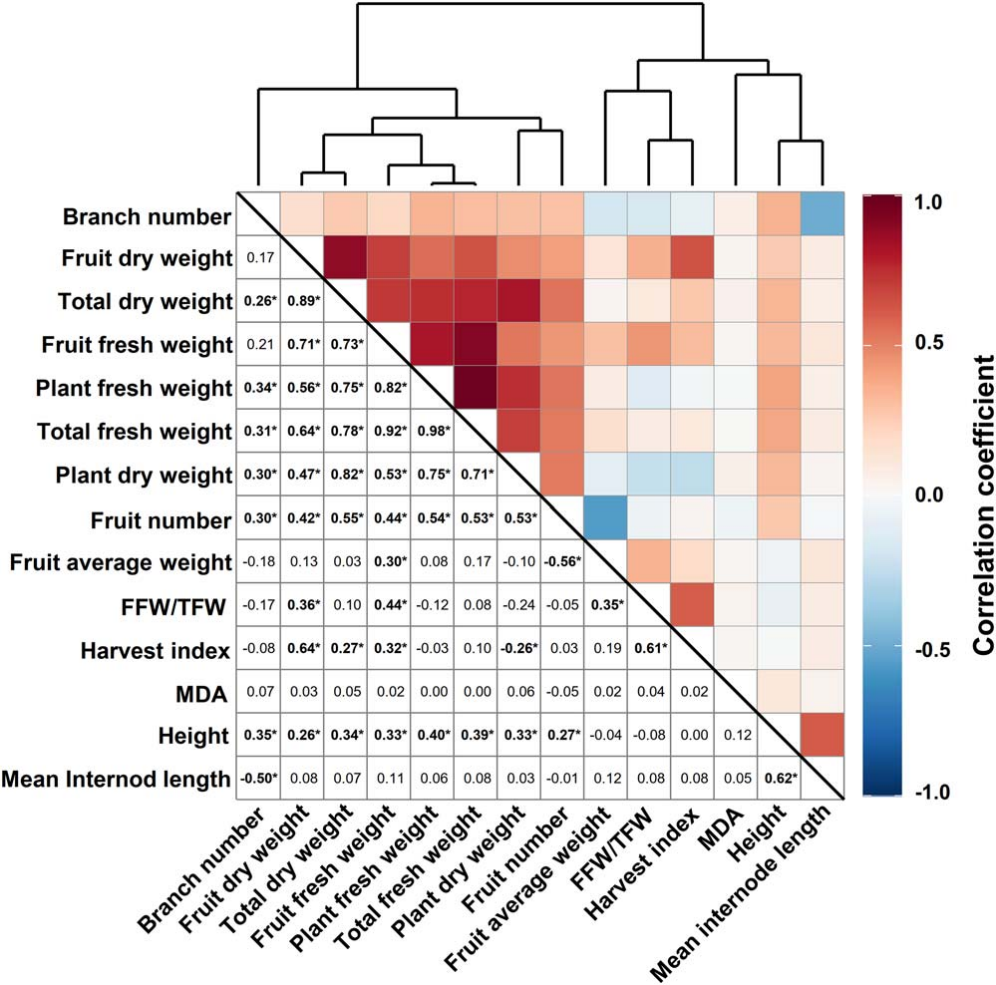
Correlation analysis of morphological traits and MDA content across the whole grafted population. The numbers in the lower triangle represent the correlation coefficient, of which the significant correlations (*q*-value <0.01) are in bold and labeled with an asterisk. FFW/TFW, the ratio of fruit fresh weight to total fresh weight.

Correlation analysis generated a cluster of significant correlations (*r* ≥ 0.42, *p* < 0.01) among seven morphological traits including FFW, FDW, PFW, PDW, TFW, TDW, and FN (Fig. 2). The strong correlation (*r* = 0.92, *p* < 0.01) between TFW and FFW resulted in relatively stable FFW/TFW across the entire population, as indicated by the lowest CV value for FFW/TFW (CV = 0.07; Fig. S2). The significant correlation between TDW and FDW (*r* = 0.89, *p* < 0.01) corroborated the stability of HI across the population with a considerably low CV (CV = 0.11, Fig. S2). HI correlated with FDW (*r* = 0.64, *p* < 0.01); however, it was only weakly correlated with TDW (*r* = 0.27, *p* < 0.01), consistent with previous findings [51, 52]. In addition, PFW was significantly correlated with FFW (*r* = 0.82, *p* < 0.01) across the population. Within each rootstock origin, the correlations between PFW and FFW remained strong (0.72 < *r* < 0.93, *p* < 0.001, Fig. S4). The consistent correlation between PFW and FFW among grafts of different rootstock origin suggests an intrinsic trait of M82 of productive efficiency, showing a trend that the bigger the final “plant size” (PFW), the higher the “fruit yield” (FFW). However, the productive ability differed between wild and domesticated rootstocks as the grafts with wild rootstocks displayed significantly lower PFW and FFW. In addition, the grafts with rootstocks from wild accessions drove a shift (*p* < 0.001) between the wild and the domesticated rootstocks on PC1, explaining 37.7% of total variation (Fig. S5a).

### Metabolic perturbation caused by rootstock diversity

**Figure 3:**
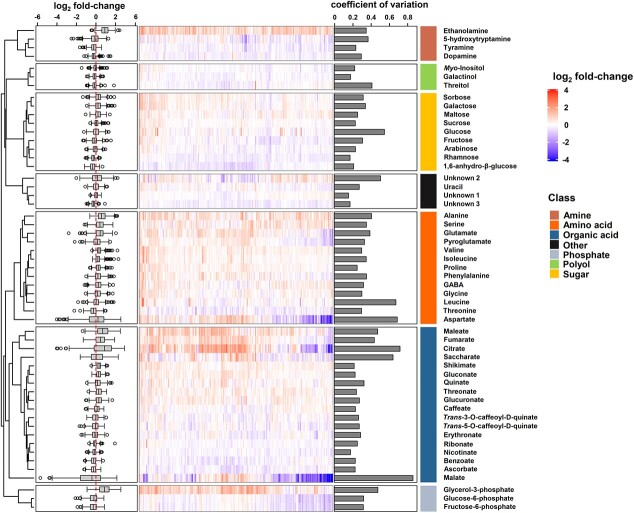
Metabolic response of scion to different rootstocks under saline conditions*.* Heatmap (middle), with a color gradient, shows the log_2_ fold-change (log_2_ FC) relative to the respective metabolites in self-grafted M82 under the saline conditions. The boxplot (left) was created based on the log_2_ FC, and the coefficient of variation (right) was calculated as the standard deviation/average based on the fold-change across the population (n = 3–4). Unknown 1: experimental RI = 1876; Unknow 2: experimental RI = 2024; Unknown 3: experimental RI = 2094.

In total, 54 metabolites were identified across 255 grafts and classified into seven classes, including organic acids, amino acids, sugars etc. (Fig. 3). To estimate variation of metabolites across the grafted population, we calculated the CV for each metabolite across the whole population. Large variability in the level of metabolites was observed across the whole population, showing a range of CVs from 0.16 to 0.86 (Fig. 3). Of all metabolites, the TCA intermediates, malate and citrate, varied greatly across the population displaying the highest CVs of 0.86 and 0.72, respectively. However, quite a few metabolites (28 of 54) were relatively stable across the population with CVs < 0.3, considered a threshold value for low variabilty [53]. Notably, “unknown 1” was the most resilient metabolite, as indicated by the lowest CV (CV = 0.16). Comparing the metabolic variations between different groups of rootstocks, we additionally observed that the metabolic variation of the SP group (CV = 0.335) was substantially higher than the SLL (CV = 0.292, *p* = 0.023) and SLC groups (CV = 0.283, *p* = 0.016) (Fig. S6). Three metabolites, citrate, malate, and aspartate with great variability, were observed as outliers, in the SLL and SLC groups, the two major transitions derived from the SP group in tomato domestication history [54, 55].

The CV analysis measured the relative dispersion and variability of each metabolite around the mean in the population of tomato grafts. Using FCs, followed by log_2_ transformation (Fig. 3), it was observed that in 70% of the grafts (n = 177) amino acids generally accumulated to a greater extent in the graft population compared to SG-M82, such as alanine, GABA, and proline etc. In particular, proline accumulation was observed in 80% of the grafts (203 of 254). A similar trend was also observed for ethanolamine, which showed a relatively low CV, but accumulated consistently in 245 grafts compared to SG-M82. Additionally, we observed that some grafts were statistically identified as outliers (positioned outside the whisker of each box plot), which contributed to the high CVs. By extracting the outliers for each metabolite, we found that specific grafts frequently appeared as outliers such as grafts 46, 51, and 54 (Fig. S7a) and were characterized by a common metabolic signature consisting of a group of six metabolites sorbose, galactose, sucrose, fructose, *myo*-inositol, and proline (Fig. S7a), that have been suggested as osmolytes against salt stress [56–58]. With regards to the effect of rootstock origins on leaf metabolism, PCA revealed that grafts of the SP group formed a cluster significantly (*p* < 0.05) distanced from other groups with the exception of the wild group on PC1, explaining 27.5% of the total variation (Fig. S5b).

### Metabolic changes across the population are associated with morphological heterogeneity

**Figure 4:**
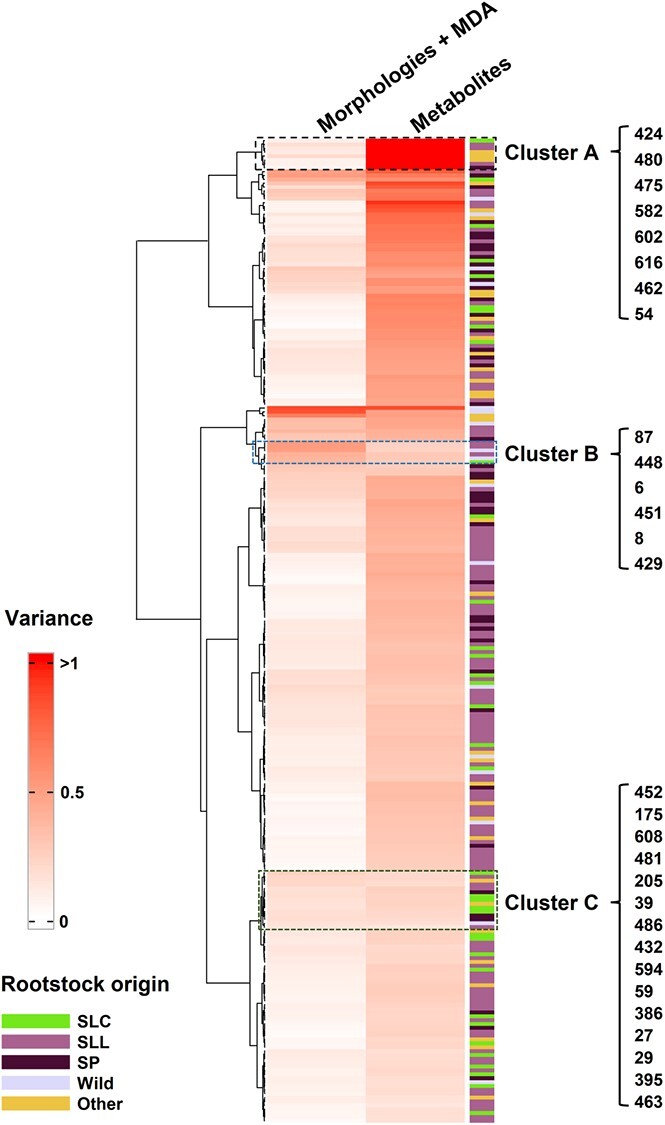
The variance of morphologies (including MDA) and metabolites of each graft relative to self-grafted M82. Abbreviations represent rootstock origin: SLC, *Solanum lycopersicum var. cerasiforme*; SL, *Solanum lycopersicum*; SP, *Solanum pimpinellifolium*; Wild, wild species; Other, accessions in processing.

By calculating the variance as described in *Equation**1*, dispersions of morphological traits, MDA, and metabolites against SG-M82 were obtained (Fig. 4), and three patterns of associations were observed. Cluster A, representing the dominant trend, typically showed relatively low variance for morphological traits (0.11 ± 0.05, n = 8) and high variance of associated metabolites (1.09 ± 0.06, n = 8) (Fig. 4). In contrast, cluster B displayed higher variance in morphological traits (0.45 ± 0.07, n = 6) than metabolites (0.26 ± 0.02, n = 6). Cluster C, as the third typical pattern, displayed quite small variance in morphological traits (0.18 ± 0.03, n = 15) and metabolites (0.20 ± 0.03, n = 15), indicating a homogenous response to the saline condition.

**Figure 5:**
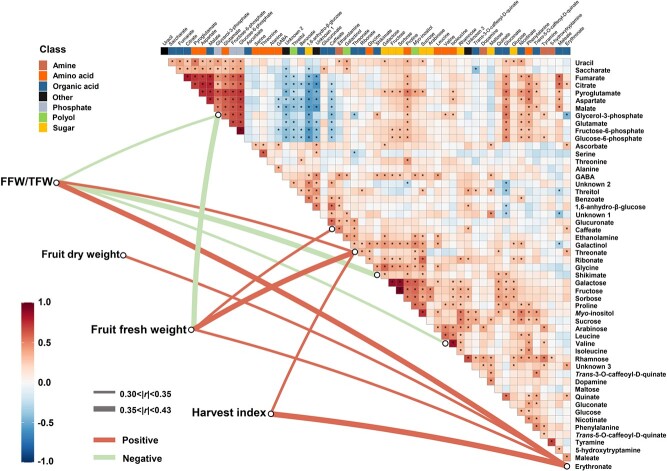
The complex correlation matrix of metabolite-metabolite and metabolite-morphology across the population using Pearson’s algorithm. The mean of fold-changes of biological replicates (n = 3–4), compared to SG-M82, was log_2_-transformed and used to construct the complex correlation matrix, consisting of metabolite-metabolite (heatmap) and metabolite-morphology (lower links). Asterisks inside the heatmap indicate *q*-value <0.05. The links filtered at a threshold of *q*-value <0.05 and |*r*| > 0.3 show the correlations between metabolites and morphological traits. The width of the edges indicates the discrete Pearson’s *r*, and the red and green colors indicate positive and negative correlations, respectively. FFW/TFW, the ratio of fruit fresh weight to total fresh weight. Unknown 1: experimental RI = 1876; Unknow 2: experimental RI = 2024; Unknown 3: experimental RI = 2094.

Next, Pearson’s correlation analysis was performed, visualized as a heatmap, to calculate metabolite-metabolite correlations and metabolite-morphology correlations (Fig. 5). A total of 340 positive and 64 negative correlations between metabolites (*q*-value <0.05 in Fig. S8, labeled with an asterisk in Fig. 5). The analysis emphasized a cluster including TCA cycle intermediates (citrate, malate), amino acids (pyroglutamate, aspartate, and glutamate), and phosphates (glycerol-3-phosphate, fructose-6-phosphate, and glucose-6-phosphate), with an *r*-value range of 0.32 to 0.93. Next to this cluster we observed a cluster of the most pronounced negative correlations between the abovementioned metabolites and a set of metabolites consisting of threitol, 1,6-anhydro-β-glucose, caffeate, and “unknown 1 and 2”. Another pattern of significant correlations was observed between the nine metabolites mentioned above and proline, gluconate, and phenylalanine. A small noticeable cluster of positive correlations comprised *myo*-inositol, proline, and sugars galactose, fructose, sucrose, and sorbose.

**Figure 6:**
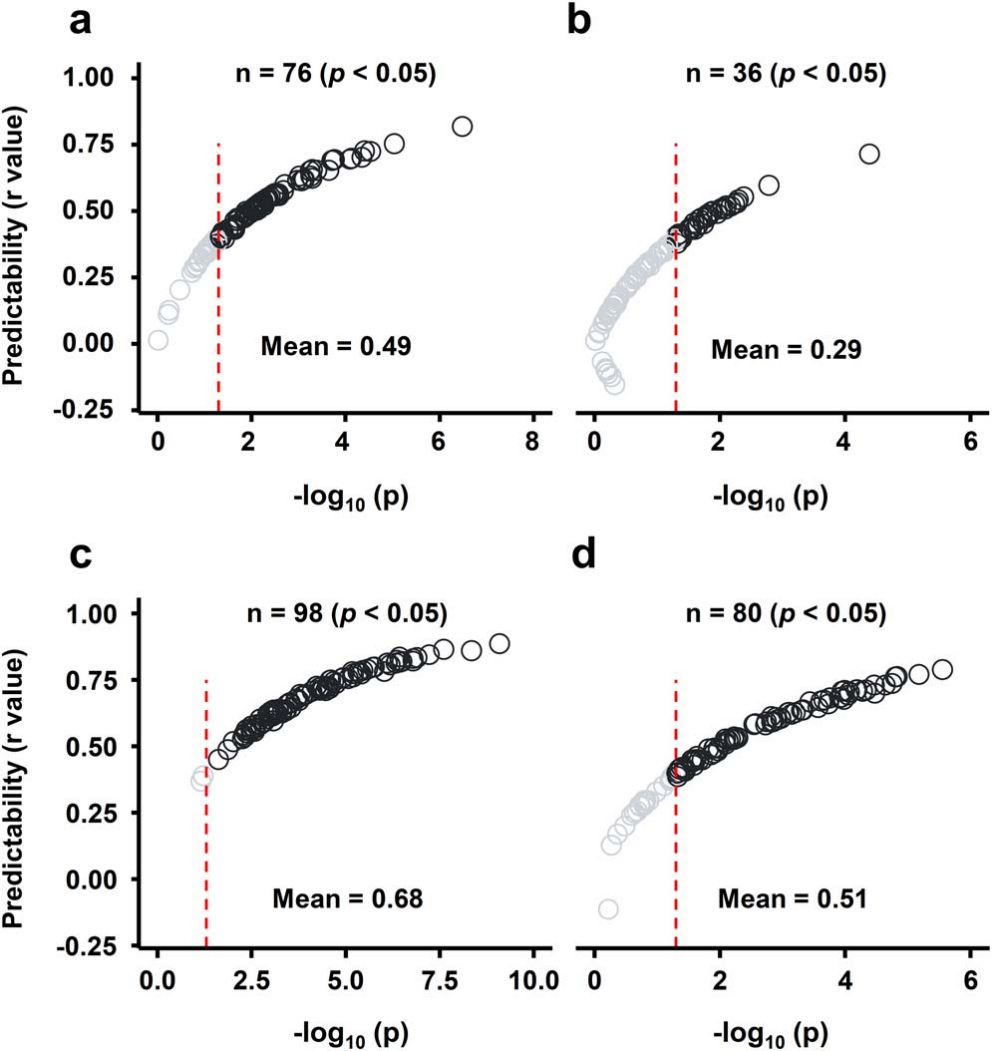
The effectiveness of predictability in metabolic prediction of the four yield-associated traits using the LASSO. **a** Fruit fresh weight (FFW); **b** Fruit dry weight (FDW); **c** The ratio of fruit fresh weight to total fresh weight (FFW/TFW); **d** Harvest index (HI). Each open circle represents one single prediction. The shaded circle indicates insignificant predictability (*p* > 0.05). The red dashed line indicates the location of the *p*-value = 0.05. The mean shows the average predictability of 100 predictions for each trait.

With a significance threshold at *p* < 0.05 and |*r*| > 0.30, correlation analysis between morphological traits and metabolites across the whole population highlighted the relationship between four yield-associated traits (FFW/TFW, FFW, FDW, and HI) and six metabolites (glycerol-3-phosphate, caffeate, threonate, shikimate, valine, and erythronate) (Fig. 5). The metabolites can be divided into two groups according to their correlations with the yield-associated traits. The first group, including caffeate, threonate, and erythronate, was positively correlated with yield-associated traits by different degrees. For instance, erythronate was associated with all four traits. However, caffeate only correlated with FFW. In the second group, consisting of glycerol-3-phosphate, shikimate, and valine, all the metabolites showed negative correlations with FFW/TFW. Among the four traits significantly correlated with metabolites, FFW/TFW displayed the most connections with all the metabolites except caffeate. In contrast, FDW was only significantly correlated with erythronate.

### Identifying putative predictive metabolic markers for yield-related traits

To capture the predictive power of a metabolite towards yield-associated traits, we used the LASSO method (Fig. 6). By performing LASSO with multiple 10-fold cross-validations, the average predictabilities of each 10-fold cross-validation were exceptionally preserved across the entire prediction process, suggesting a homogeneous distribution for sample partition and the reliability for variable selection (Fig. S9, Table S4). FFW/TFW was regarded as the trait with the highest predictability, displaying average predictability of 0.68 (Fig. 6), followed by the predictability of HI (0.51) and FFW (0.49). FDW was the trait with the lowest predictability (0.29, Figs. S10 and S11).

**Figure 7:**
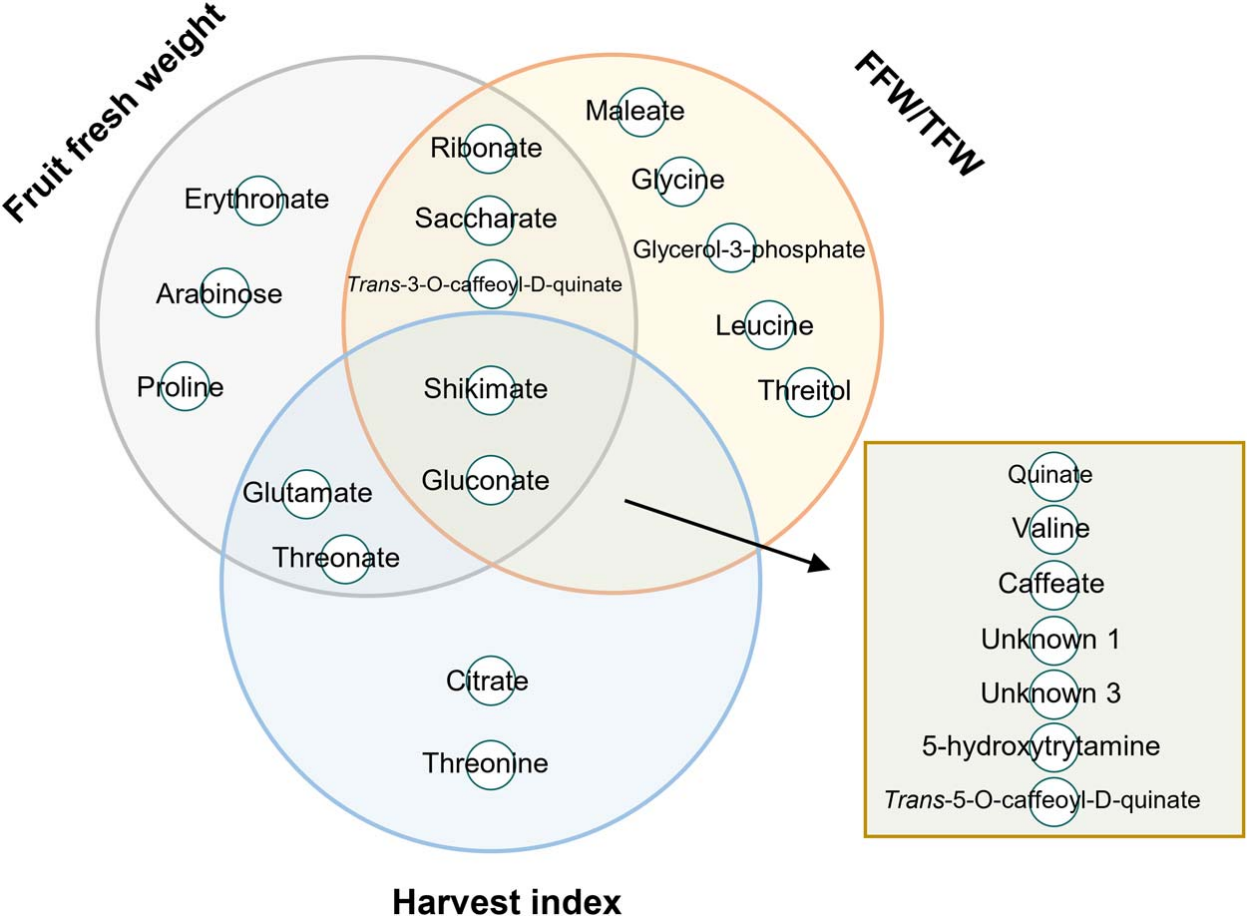
The Venn diagram of the overlap of frequently selected metabolites (n ≥ 95) between the predictions of fruit fresh weight, harvest index, and FFW/TFW (the ratio of fruit fresh weight to total fresh weight). Unknown 1: experimental RI = 1876; Unknown 3: experimental RI = 2094.

Instead of using all metabolites, LASSO performed variable selection to improve predicting accuracy [59]. This enabled us to investigate the groups of metabolites contributing to the prediction of each trait. LASSO selected a set of “important” metabolites, from the 54 annotated metabolites, in each prediction for each trait, forming a list of frequently selected metabolites from 100 predictions (Table S4). Metabolites with stronger predictive values are more likely to be selected in each prediction test. In the merged list of frequently selected metabolites from the prediction of four traits, the most predominant metabolite groups were organic acids such as citrate, shikimate, and quinate, and amino acids such as glutamate, glycine, and leucine, accounting for 50% and 25% of the frequently selected metabolites, respectively. Among the frequently selected metabolites, gluconate and shikimate were observed to predict FFW, FFW/TFW, and HI with a frequency of at least 99 times (Table 1, Fig. 7). In addition, three, five, and two metabolites were specifically frequently selected for the prediction of FFW, FFW/TFW, and HI, respectively (Fig. 7).

## Discussion

### Grafting exposes a broad spectrum of changes in morphological traits and MDA content under saline conditions

A population of grafted tomato plants was generated in which the unitary shoot from commercial variety M82 (*Solanum lycopersicum*) was grafted onto rootstocks of 254 accessions, representing various groups across the domestication history of tomato, consisting of wild species (Wild), *Solanum pimpinellifolium* (SP), *Solanum lycopersicum var. cerasiforme* (SLC)*, S. lycopersicum* L. var. *lycopersicum* (SLL), and few other accessions (Other). Among the different grafts, the genetic background of the rootstocks used is the sole factor influencing M82 scion growth and development under saline conditions.

Following 34 days of growth, phenotypic diversification was evident. The oxidative stress marker MDA exhibited the highest variability (CV = 0.58), while FFW/TFW was the most conserved trait in the collection. FFW/TFW and HI, which represent the ability of a plant to allocate assimilated photosynthates to the harvestable product [60, 61], were inherently resilient and strongly dependent on the M82 scion. The significant correlation between FFW and PFW suggests that the bigger the plant (higher PFW), the greater the fruit yield (FFW). Our results also suggest intrinsically robust productivity of M82, irrespective of rootstock origin. Based on this notion, it can be expected that vigorous rootstocks are likely to improve the FFW of grafted plants [62].

The phenotypic heterogeneity mediated by rootstocks has only been fragmentarily documented in grafted tomatoes under standard growth conditions [63] and non-optimal environments [30, 64, 65]. Mauro et al. reported the contributions of rootstock origins to scion growth [66]. The grafts with a rootstock of *Solanum habrochaites*- and *S. pimpinellifolium*-derived hybrids showed a reduction in fruit biomass, but two hybrids had opposite effects on plant biomass under optimal conditions. Our study shows that rootstock-mediated phenotypic diversification is expressed differently across the measured morphological traits under non-optimal conditions of growth (Fig. 1).

Although different domesticated transitions were tested, similar dispersion on PCA plots was noticed between the relatively close transitions SLL, SLC, and SP, when the analysis was built using morphological traits (Fig. S5a). In contrast, the grafts with wild species as rootstocks showed a significant impact on the dispersion across PC1. These results indicate that human selection led to a relatively homogeneous adaptive trait to suboptimal growth conditions [67, 68].

### Metabolic variation suggests a shift in carbon allocation towards stress metabolism across the graft population

Metabolite profiling across the collection suggests that the differences between grafts were only marginally affected by rootstock origin (Fig. S4b). That said, metabolites displaying a significant response comprised the major organic acids, citrate, malate, and the amino acid, aspartate, particularly in SLL and SLC groups rather than in SP group. Considering the domestication history of SP, SLL, and SP groups [54, 55, 69], our results suggest that the domestication process likely boosted the performance of modern tomato cultivars by modulating central energy-associated metabolites. Across the profile, the relative content of malate, citrate, and fumarate changed in association with individual rootstocks (Figs. 3 and 5), potentially indicating that under non-optimal growth conditions, rootstocks can mediate central pathways in carbon metabolism [70]. Sub-optimal conditions can cause a reduction in plant assimilation as well as an increase in the energy cost of stress defense [71], leading to reduced plant growth rate due to greater respiration for the plant maintenance [72, 73]. Contrary to expectations, no strong (|*r*| > 0.3) or significant correlation (*q* < 0.05) were obtained between traits related to plant growth and energy-related metabolites (Fig. 5), suggesting an indirect link between central carbon metabolism and plant growth. Carbohydrates are highly associated with source-to-sink carbon partitioning in tomato [74]. That said, energy metabolism is balanced via the regulation of the TCA cycle in mitochondria [75] and by replenishment of TCA intermediates from amino acids [76]. Our analysis revealed the accumulation of amino acids in most of the grafts (177 out of 254) compared with SG-M82 (Fig. 2). For instance, among these amino acids, the metabolism of GABA plays an essential role in nitrogen and carbon metabolism under stressed conditions [77, 78].

Plants under salt stress can accumulate compatible osmolytes such as proline, sucrose, and *myo*-inositol in the cytoplasm to maintain osmotic potential when Na^+^ is sequestered in the vacuole to avoid the deleterious effects of Na^+^ and Cl^−^ on metabolic process [56, 79, 80]. The accumulation of osmolytes may be the consequence of the shift of energy consumption into stress defense [81]. We observed a noticeable cluster of positive correlations among osmolytes including *myo*-inositol, proline, and the sugars galactose, fructose, sucrose, and sorbose (Fig. 5), indicative of the coordinating role of these metabolites under saline conditions, and showing that this response is generally conserved between grafts. The six osmolytes were also significantly correlated with metabolites such as glycerol-3-phosphate, fructose-6-phosphate, and glucose-6-phosphate (Fig. 3), which have a role in energy supply and osmotic adjustment [79, 82]. For example, it has been documented that the enhancement in salt tolerance of tomato plants is linked to the overexpression of the chloroplast glycerol-3-phosphate acyltransferase gene (*LeGPAT*) [83].

### Correlation analysis highlights the relation between yield-associated traits and leaf central metabolism

Using the 54 annotated metabolites and 14 morphological traits (including MDA content) in scions across the graft population, we calculated the overall variance, correlation distribution, and implemented model prediction to reveal associations between metabolites and morphological traits. The variance-based analysis indicated the existence of three major groups of grafts (Fig. 4) differing in modulation of their traits. For instance, cluster A showed relatively low variance for morphological traits while displaying high variance of associated metabolites. These data suggest that the plant phenotypes were robustly maintained by extensive alteration of central metabolism.

The correlation analysis identified significant relations between four yield-associated traits and six metabolites (|*r*| >0.3, *q* < 0.05, Fig. 3). These are relatively sparse relations compared to earlier analysis on interspecific tomato introgression lines [43] and might reflect a more complex effect of rootstocks on scion growth. Specifically, among the six metabolites, glycerol-3-phosphate is associated with energy supply related to salt stress [82, 83], threonate is the end-product of ascorbate degradation [84, 85], and caffeate has been linked with different stress responses due to its role in the basic metabolic process of lignin synthesis [86]. Among the yield-associated traits, FFW/TFW and HI, which represent the efficiency of partitioning of assimilated photosynthate to harvestable product, showed no link with central energy metabolites (Fig. 3). In common with findings from Schauer et al. [43], the metabolites that correlated strongly with yield-associated traits seem to be more stable across the population, showing relatively low CVs (Figs. 3, S2*)*.

**Table 1:** The summary of frequently selected metabolites (frequency ≥ 95) in the metabolic prediction of yield-associated traits. The result for fruit dry weight is shaded due to its ineffective prediction. FFW, fruit fresh weight; FDW, fruit dry weight; FFW/TFW, the ratio of fruit fresh weight to total fresh weight; HI, harvest index

**Class**	**Metabolite**	**FFW**	**FDW**	**FFW/TFW**	**HI**
**Amino acid**	Glutamate	100	-	-	98
	Glycine	-	-	100	-
	Leucine	-	-	100	-
	Proline	100	-	-	-
	Threonine	-	-	-	97
	Valine	-	-	100	100
**Organic acid**	Citrate	-	-	-	100
	Shikimate	100	96	100	100
	Quinate	-	-	100	100
	Ribonate	97	-	100	-
	Saccharate	96	-	97	-
	Caffeate	-	96	98	100
	Gluconate	100	-	100	99
	Erythronate	100	100	-	-
	Threonate	100	-	-	100
	Maleate	-	-	95	-
	*Trans*-3-O-caffeoyl-D-quinate	99	-	97	-
	*Trans*-5-O-caffeoyl-D-quinate	-	95	99	100
**Sugar**	Arabinose	98	-	-	-
**Phosphate**	Glycerol-3-phosphate	-	-	98	-
**Amine**	5-hydroxytryptamine	-	-	100	100
**Polyol**	Threitol	-	-	100	-
**Other**	Unknown 1^*^	-	-	100	100
	Unknown 3^*^	-	-	100	100

* Unknown 1: experimental RI = 1876; Unknown 3: experimental RI = 2094.

### Leaf metabolites are associated with FFW/TFW, HI, and FFW with predictive potential

The association between plant phenotype and metabolism is complex due to the intricate system(s) of different regulatory levels [87]. Various prediction models, addressing the non-linear relationship between trait expressions and predictors [88–90], have been used in an effort to link phenotypes in different species, such as rice [91], cotton [92], maize [93], and wheat [94]. Most reports have examined the relationship between plant traits and “omics” data of populations grown under optimal conditions [45, 95, 96]. The LASSO method, combining both shrinkage and variable selection methods [90], was shown to be quite efficient in metabolic prediction of yield traits [45]. Here, we investigated the association between scion growth features and leaf metabolomics data using the LASSO method based on the results of our correlation analysis. The model yielded effective predictions for FFW/TFW (*r* = 0.68), HI (*r* = 0.51), and FFW (*r* = 0.49) (Fig. 6), validated by a permutation test at empirical *p* < 0.05 (Fig. S10), revealing the great importance of metabolites for predicting traits.

Usually, model prediction has been performed on a collection consisting of subpopulations, for instance, from different species [93], generations [88], and years [97]. However, the accuracy and efficiency of predictions can be affected by the genetic distance in populations [98, 99]. In the present study, PCA plots showed an admixture of grafts from different rootstock origins on phenotypes and metabolites (Fig. S5) and avoided the effect of subpopulation structures in model prediction. Besides the effect of population structure, the sample partitioning in the datasets for model training and testing plays a vital role in prediction [88]. 10-fold cross-validation was widely used in genomic selection for evaluating the ability and efficiency of the prediction model [90]. The typical 10-fold cross-validation was applied ten times to partition the population for model training and testing and exhibited plateaued predictabilities of FFW/TFW, HI, and FFW, indicating high prediction accuracy (Fig. S9).

The concern exists for the practical application of the metabolite contributed prediction of phenotypic traits, as these profiles capture a snapshot of the highly dynamic plant metabolism system(s) that change substantially over time and conditions [100]. The highly predictive model for the traits based on the metabolite profiles from a specific time point may not be applicable for predicting traits over different time points of plant growth [46]. Here, we performed metabolite profiling on leaflet samples from all grafts at the same development stage and under the same conditions of growth to address the indirect links between morphologies and metabolites. Our findings may be implemented with samples from different time points of plant growth to gain a broader knowledge of metabolite-mediated scion performance in future study.

Moreover, instead of using unknown metabolic features as predictors [45, 101], we used 54 annotated metabolites, which greatly facilitated understanding of the metabolic contribution to trait expression. Metabolites, as intermediates and end-products of biochemical pathways, can have close connections with phenotypes [43, 102]. Correlation analysis between the levels of metabolites and phenotypes has been used to estimate the conceivable function of a metabolite in the modulation of a phenotype [103, 104]. However, it is assumed that a single metabolite generally exerts only a moderate or no impact on trait expression [89]. Either in metabolic or genetic prediction, the LASSO method enables metabolites to be identified with high predictive potential [45]; For example, four out of five metabolites displaying significant correlation with FFW/TFW were frequently selected (frequency ≥ 90) in prediction, including glycerol-3-phosphate, shikimate, valine, and threonate (Fig. 5, Table S4). However, the list of frequently selected metabolites in the prediction of HI only comprised threonate (frequency = 90), though threonate significantly correlated with erythronate (Fig. 5). This may be explained by that LASSO may select the predictor with powerful complementary information [59]. The predictor selection via LASSO model showed the applicability of predicting yield-associated traits using leaf metabolic profiling data, as evidenced by the overlaps of the frequently selected metabolites between traits (Fig. 7). Especially, the frequently selected metabolites, suggest their pivotal role in the modulation of the corresponding trait. For instance, the TCA intermediate citrate, specifically for HI, may indicate its central role in the carbohydrate partitioning in plant between the vegetative and the reproductive organs, as described above.

In conclusion, we have shown the phenotypic diversity of scion and metabolic perturbation in leaves, which were both modulated by rootstocks in response to saline conditions. We found highly responsive trait (MDA) and intrinsic traits (FFW/TFW and HI) of M82 across the graft population. Leaf metabolites malate, citrate, and aspartate showed to be central in the response to salinity and in the rootstock-mediated energy repartitioning between plant growth and stress defense. The indirect connections between morphology traits and metabolite content were complemented and expanded with a predictive model LASSO, which emphasized the role of metabolites in phenotype modulation. Future studies should tackle the regulatory mechanisms underlying these associations. Our results could provide new insights for further research in grafting biology in relation to abiotic stress and set the basis for metabolic marker assisted selection of rootstock mediated tolerance to salinity.

## Materials and methods

### Plant materials

A total of 254 tomato accessions were collected (Table S1) including 45 accessions of *S. pimpinellifolium* L. (SP), 36 *S. lycopersicum var. cerasiforme* (SLC), 122 *S. lycopersicum* L. var. *lycopersicum* (SLL), 34 accessions which are still in processing (Other), and 17 accessions of wild species (Wild) including *Solanum chmielewskii*, *S. habrochaties*, *S. huavlasense*, *S. peruvianum*, *S. corneliomuelleri*, and *Solanum neorickii*. The shoot of commercial *S. lycopersicum* cv. M82 was used as a scion and grafted onto the rootstocks of the 254 accessions. The self-grafted M82 (SG-M82) was used as the reference for comparison with other grafts. For grafting, 30 day-old tomato seedlings were used to establish tomato grafts; in total 980 grafted plants consisting of 3 to 4 biological samples for each accession.

### Experimental setup

The experiment was conducted in August–October 2017 in a plastic net house, located on the Sede Boqer campus of Ben-Gurion University, Israel (30°52′08.04″N, 34°47′33″E, altitude 480 m). Twenty days after grafting, plants were transplanted into 10-L growth pots with washed sand. Following an 18-day adaptation period, all plants were subjected to irrigation with a saline solution (200 mM NaCl +0.5 g/L commercial fertilizer solution), yielding a final EC of 20 dS m^−1^. NaCl concentration was gradually increased from 0 to 200 mM over a 9-day period to avoid osmotic shock (measured salinity in the irrigation solution was 50 mM on days 1–2, 100 mM on days 3–4, 150 mM on days 5–8, and 200 mM on day 9). The amount of leachate was maintained at ~20% of the irrigation solution with the aim of avoiding salt accumulation in the sand. In addition, every 7 days, pots were washed using an automatic drip irrigation system with a 2-L solution (200 mM NaCl +0.5 g/L fertilizer) to avoid salt accumulation. On a regular basis, plants were irrigated with a 0.5–1.5 L, adjusted to the developmental stage, i.e. from vegetative to mature fruiting stage [105], every morning (7:30 am, local time, GMT + 3) throughout the experiment. The aboveground parts of the plants were harvested 40 days after treatment application.

### Morphological measurement

Thirteen morphological traits were measured on the aboveground tissue (Table S2): plant height (PH, cm) was determined from the ground to apex of main shoot; branch number (BN) and fruit number (FN) were counted for each plant; mean internode length (MIL, cm) was calculated as PH/BN; fruit fresh weight (FFW, g plant^−1^) was determined from the total weight of red and green fruits; mean fruit weight (MFW, g fruit^−1^) was calculated as FFW/FN; plant fresh weight (PFW, g plant^−1^) was determined by weighing the aboveground vegetative tissue without fruits; total fresh weight (TFW, g plant^−1^) was calculated as the sum of FFW and PFW; fruit dry weight (FDW, g plant^−1^) and plant dry weight (PDW, g plant^−1^) were obtained by drying fruits and vegetative tissue, respectively, until reaching constant weight; total dry weight (TDW, g plant^−1^) was determined as the sum of FDW and PDW; the ratio of fruit fresh weight to total fresh weight (FFW/TFW) was calculated; harvest index (HI) was calculated as FDW to TDW.

### Estimation of malondialdehyde (MDA) in tomato leaflets

Leaflets collected on day 35 were used for MDA estimation as described previously [106] with some modifications. Briefly, tomato leaf tissue (40 mg) was homogenized using a retch mill, pre-cooled with liquid nitrogen and then resuspended in ice-cold extraction mixture (1:20, mg FW: μL) followed by centrifugation at 8000 rpm for 10 min (Eppendorf, Germany). The extraction mixture was composed of phosphate buffer saline (PBS), 0.1 mM phenylmethanesulfonyl fluoride (PMSF), and 10% (w/v) trichloroacetic acid. The supernatant was transferred to a new 2-mL Eppendorf tube and mixed with one equivalent volume of 0.8% (w/v) thiobarbituric acid (TBA). After mixing vigorously, samples were heated at 90°C for 45 min, cooled, and centrifuged at 1000 rpm for 15 min (Eppendorf, Germany). Absorbances at 532 nm and 600 nm were measured in an Epoch Microplate Spectrophotometer (BioTek) using Gen5 2.05 software to calculate equivalent MDA content (nmol g^−1^ FW). To avoid variation from different measurement periods, a reference sample (SG-M82) was mixed, equally aliquoted, and arranged into each batch of measurements. Thus, the levels of MDA are presented as fold-change over SG-M82 (Table S2).

### Sample preparation for gas chromatography–mass spectrometry (GC–MS) analysis

Sampling of the youngest fully-expanded leaflet for GC–MS analysis was carried out on day 34 following the recommended metabolite data reporting protocol [107]. Leaflet samples were frozen in liquid nitrogen and freeze-dried. Lyophilized leaflets were ground into powder using a pre-cooled mixer mill (MM 400, Retsch, Haan, Germany) with two steel beads at 25 Hz for 2 min. Metabolite extraction was performed with a few modifications based on the previous research [108]. Briefly, 25 mg of tissue powder was extracted in 1.28 ml of a pre-cooled extraction mixture containing chloroform: methanol: water (2.5: 1: 1, v/v). The mixture was briefly vortexed and incubated on an orbital shaker (Keison, UK) at 25°C at 1000 rpm for 10 min. The mixture was sonicated at room temperature for 10 min and then centrifuged at 20000 g for 10 min (Eppendorf, Germany). The supernatant (900 μl) was transferred to a fresh 2-mL Eppendorf tube, and 300 μl each of chloroform and water were added. Phase separation was achieved by centrifuging the mixture at 20000 g for 10 min. The supernatant of each sample was collected and stored at −80 before GC–MS analysis. 50 μl of supernatant from each sample was pooled to generate a quality control (QC) for data normalization.

### Metabolite profiling

A GC–MS (7890B-5977B, Agilent, Santa Clara, CA) with a setup as described previously [109] was used for the relative quantification of non-targeted metabolic features in leaf samples. Metabolite annotation was validated using a Mass-hunter Workstation 8.0 (Agilent Technologies, US) based on spectral searching supported by the National Institute of Standards and Technology (NIST, USA) against RI libraries from the Max Plank Institute for Plant Physiology (Golm, Germany). To remove the batch effect due to long-term GC–MS running, a freely available normalization method, Systematic Error Removal using Random Forest (SERRF) [110], was performed based on the QC samples, which were run along with experimental samples. The final levels of detected metabolites, referred to as relative content, were based on the peak area of the selected mass, which was obtained and normalized to the corresponding metabolite in QC sample (Table S3).

### Metabolomics-based prediction of yield-related traits in the grafted population

The Least Absolute Shrinkage and Selection Operator (LASSO) [111] implemented in the “glmnet” package [112] was used for the metabolic prediction of morphological traits. LASSO was applied using the metabolic data of 54 annotated metabolites to predict each of the four yield-associated traits derived from the morphology-metabolite correlation analysis. To evaluate the ability of the model built by LASSO to predict data not used to train the model, a 10-fold cross-validation scheme was used (Fig. S1). For cross-validation, the population of tomato grafts was randomly divided into ten subsets with an approximate equal sample size. Metabolic and morphological data of the nine sets were used for model training, performing another 10-fold cross-validation to obtain tuning parameters, and the metabolic data of the remaining tomato grafts were applied to the trained model to obtain the predicted value of the morphological trait. Thus, the model testing was repeated ten times so that each population set could be included. To exclude partitioning dependence of the predictive test, we repeated the 10-fold cross-validation ten times, each randomly partitioning 255 accessions into ten new subsets. The strength of predictability is given by the correlation coefficient between the predicted and the observed values of the morphological trait and the corresponding *p*-value. This resulted in 100 predictabilities from 100 predictions for each trait. A permutation test was performed to assess the statistical significance of the observed predictability from the null distribution. In the permutation, the shuffled morphological data were assigned to the samples for prediction, repeated 100 times. The null distribution, formed from 10 000 permutation tests, consisting of 100-times permutation tests for each prediction, was compared against the mean of predictabilities to calculate an empirical *p*-value for each trait, as suggested from previous research [113].

### Data processing and statistical analysis

To evaluate the variation of morphologies and metabolites across the grafted population, the fold-change (FC) was calculated as the mean of biological replicates of each graft divided by that of SG-M82 under the same saline condition for each morphological trait and metabolite.

For morphology-morphology, metabolite-metabolite, and metabolite-morphology correlation analyses, log_2_-transformed fold-changes (log_2_ FCs) of morphological traits and metabolites were used. For correlation analysis between tomato grafts, the mean values of morphological traits and metabolic data were normalized to the median of the population for each trait and metabolite, followed by log_2_-transformation, respectively. All correlation analyses were performed using the *corr.test* function and “Pearson” algorithm provided in the “pysch” package [114]. Visualization of the correlation matrix was achieved using the *corrplot* function in the “corrplot” package [115] using the Ward.D2 clustering method in the *hclust* function built in the “factoextra” package [116].

A clustered heatmap with annotations was created based on log_2_ FCs using the *Heatmap* function within the “Complexheatmap” package [117]. Principal component analysis (PCA) was performed using SIMCA 14.1 (Umetrics, USA) and visualized using the “ggplot2” package [118]. The diagram was created using the online tool “Venny” version 2.1.0 [119]. Statistical analysis was performed using “R” platform version 3.6.3 [120] and JMP®, version 13 (SAS Institute Inc., Cary, NC, 1989–2007). The functions *t.test*, *Wilcox.test*, and multiple comparisons using Tukey’s HSD test were used in corresponding comparisons between grafts according to the data distribution.

Variance in probability theory and statistics, measures the distance that a set of numbers is spread around the average value. The log_2_ FC, relative to SG-M82, normalized the distance of the values of different traits (morphological and metabolic) from SG-M82. To measure the dispersal of traits of each graft around the value of SG-M82, we used the modified variance measure:

(1) \begin{equation*} \mathrm{Var}\left(\mathrm{X}\right)=\frac{1}{n-1}{\sum}_{i=1}^n{\left({\log}_2{\mathrm{FC}}_i-\mathrm{Con}\right)}^2 \end{equation*}

where:

Var(*X*) = Dispersal of traits or metabolites of graft X around control.

${\log}_2{FC}_i$ = log_2_-transformed fold changes relative to SG-M82 for trait *i* or metabolite *i*.

Con = log_2_ FC of control, it is constant zero.

N = The number of morphological traits including MDA or metabolites.

## Acknowledgments

The work was supported by the Israeli Ministry of Agriculture and Rural Development (REA-NET-5317) as part of the project The Root of the Matter: roots knowledge center for leveraging modern agriculture. We thank Dani Zamir for kindly providing us tomato seeds for this experiment. We greatly thank Dr. Kelem Gushu, Dr. Maria Dolores, Dr. Moses Kwame, Dr. Noam Reshef, Yaara Zohar, Noga Sikron, Lina Zhao, Liron Summerfield, and Junyi He for their support in the field and lab.

## Author Contributions

C.S., T.A., and M.A.A carried out the experiment, plant harvesting, and leaf sampling. C.S. and T.A. were responsible for MDA estimation and metabolic extraction. C.S. performed GC–MS analysis, interpreted data, and wrote the manuscript. S.B., N.L, and A.F. designed this experiment, supervised the work, and revised the manuscript.

## Data Availability

The data and materials supporting the conclusions of this study are included in supplementary information.

## Conflicts of interest statement

The authors declare no competing interest.

## Supplementary data

Supplementary data is available at *Horticulture Research* online.

## Supplementary Material

Web_Material_uhac061
