# Supplementary material for: Leveraging a graft collection to develop metabolome-based trait prediction for the selection of tomato rootstocks with enhanced salt tolerance
Source: Hortic Res. 2022 Mar 14;9:uhac061. doi: 10.1093/hr/uhac061 (PMC9071376; doi:10.1093/hr/uhac061)
Supplement: Web_Material_uhac061 [file web_material_uhac061.zip › supplementary reference.docx]

**Supplementary reference**

Birkemeyer, C. & Kopka, J. Design of metabolite recovery by variations of the metabolite profiling protocol. In: Nikolau B.J., Wurtele E.S. (eds) Concepts in plant metabolomics. Springer, Dordrecht. 45-69 (2007).

De Miguel, M. *et al.* Organ-specific metabolic responses to drought in Pinus pinaster Ait. *Plant Physiol. Biochem*. **102,** 17-26 (2016).

Gupta, P. & De, B. Metabolomics analysis of rice responses to salinity stress revealed elevation of serotonin, and gentisic acid levels in leaves of tolerant varieties. *Plant Signal Behav*. **12,** e1335845 (2017).

Kalozoumis, P. *et al.* Impact of plant growth-promoting rhizobacteria inoculation and grafting on tolerance of tomato to combined water and nutrient stress assessed via metabolomics analysis. *Front. Plant Sci*. **12,** 670236 (2021).

Kiani-Pouya, A. *et al.* Epidermal bladder cells confer salinity stress tolerance in the halophyte quinoa and Atriplex species. *Plant Cell Environ*. **40,** 1900-1915 (2017).

Li, M.Q. *et al.* Melatonin mediates selenium-induced tolerance to cadmium stress in tomato plants. *J. Pineal Res*. **61,** 291-302 (2016).

Li, Y. *et al.* MicroTom metabolic network: Rewiring tomato metabolic regulatory network throughout the growth cycle. *Mol. Plant*. **13,** 1203-1218 (2020).

Negri, S., Commisso, M., Avesani, L. & Guzzo, F. The case of tryptamine and serotonin in plants: a mysterious precursor for an illustrious metabolite. *J. Exp. Bot*. **72,** 5336-5355 (2021).

Nunes-Nesi, A. *et al.* Identification and characterization of metabolite quantitative trait loci in tomato leaves and comparison with those reported for fruits and seeds. *Metabolomics*. **15,** 46 (2019).

Shabala, L. *et al.* Cell-type-specific H^+^-ATPase activity in root tissues enables K^+^ retention and mediates acclimation of barley (*Hordeum vulgare*) to salinity stress. *Plant Physiol*. **172,** 2445-2458 (2016).

Tannert, M. *et al.* Pi starvation-dependent regulation of ethanolamine metabolism by phosphoethanolamine phosphatase PECP1 in Arabidopsis roots. *J. Exp. Bot*. **69,** 467-481 (2018).

Terce-Laforgue, T. *et al.* Resolving the role of plant NAD-glutamate dehydrogenase: III. overexpressing individually or simultaneously the two enzyme subunits under salt stress induces changes in the leaf metabolic profile and increases plant biomass production. *Plant Cell Physiol*. **56,** 1918-29 (2015).

Ye, J. *et al.* Genome-wide association analysis identifies a natural variation in basic helix-loop-helix transcription factor regulating ascorbate biosynthesis via D-mannose/L-galactose pathway in tomato. *PLoS Genet*. **15,** e1008149 (2019).

Zhu, G. *et al.* Rewiring of the fruit metabolome in tomato breeding. *Cell*. **172,** 249-261 (2018).
