# Supplementary figures and images for: Leveraging a graft collection to develop metabolome-based trait prediction for the selection of tomato rootstocks with enhanced salt tolerance
Source: Hortic Res. 2022 Mar 14;9:uhac061. doi: 10.1093/hr/uhac061 (PMC9071376; doi:10.1093/hr/uhac061)

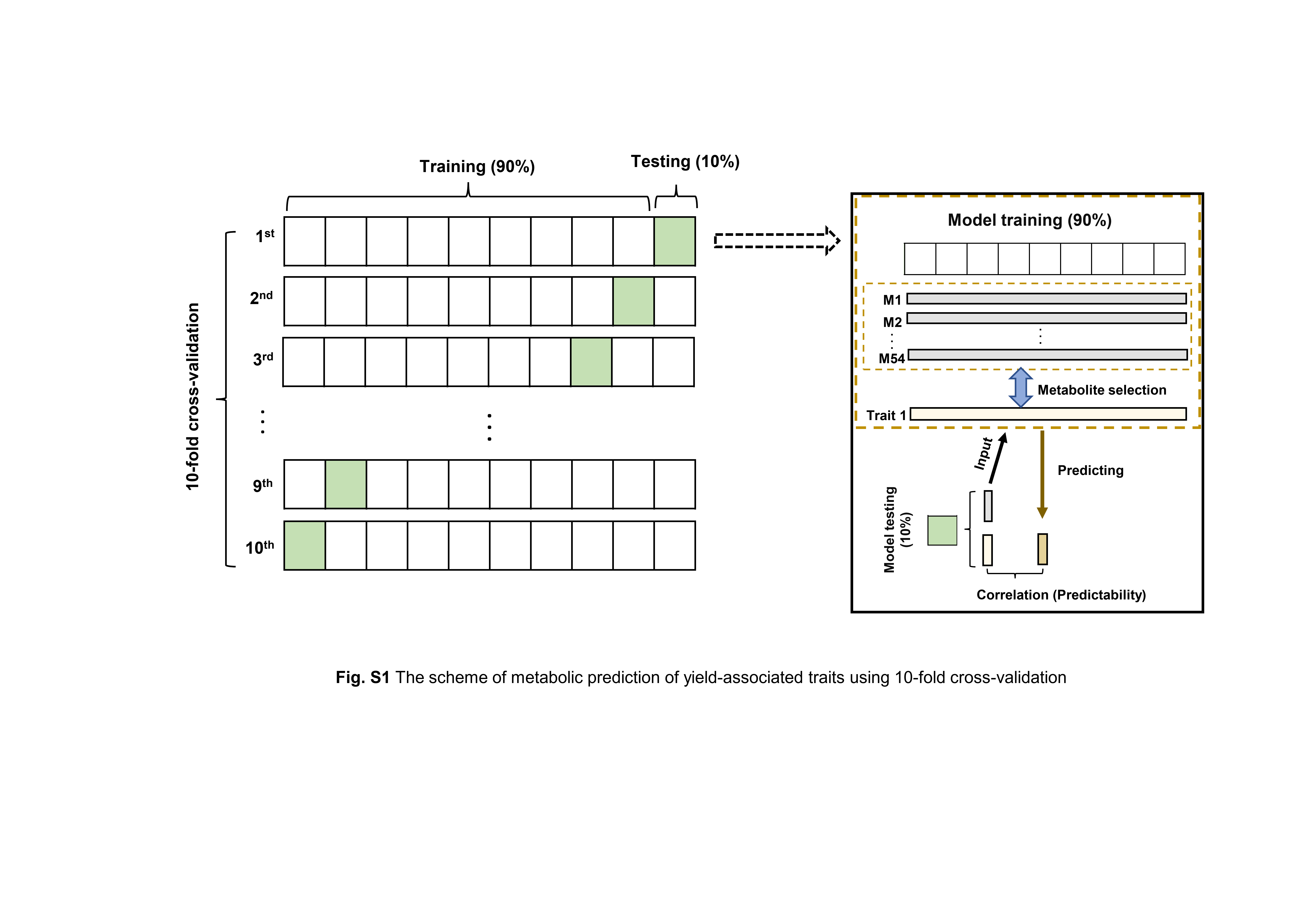

Supplement: Web_Material_uhac061 [file web_material_uhac061.zip › Fig. S1.tif]

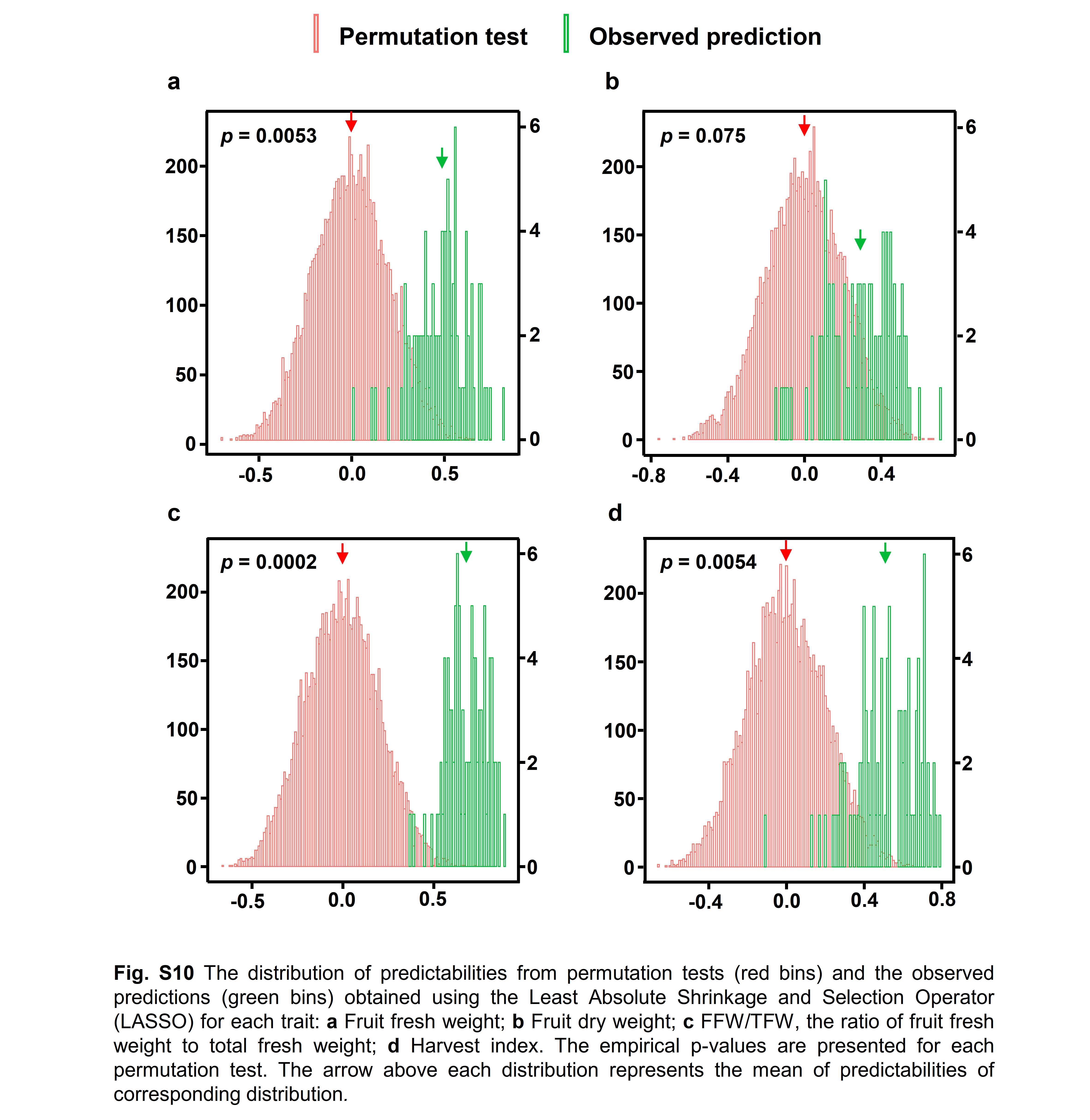

Supplement: Web_Material_uhac061 [file web_material_uhac061.zip › Fig. S10.tif]

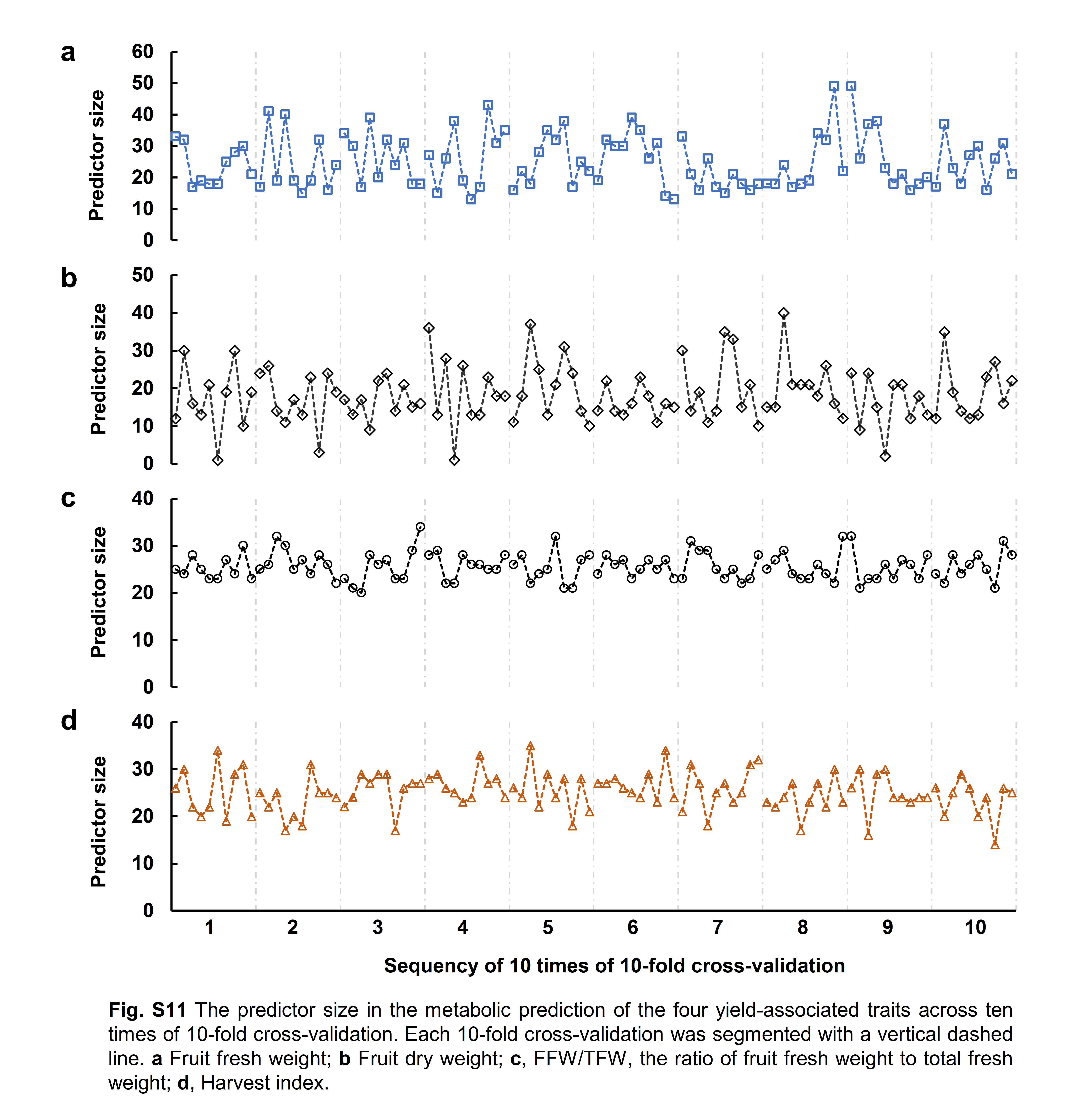

Supplement: Web_Material_uhac061 [file web_material_uhac061.zip › Fig. S11.tif]

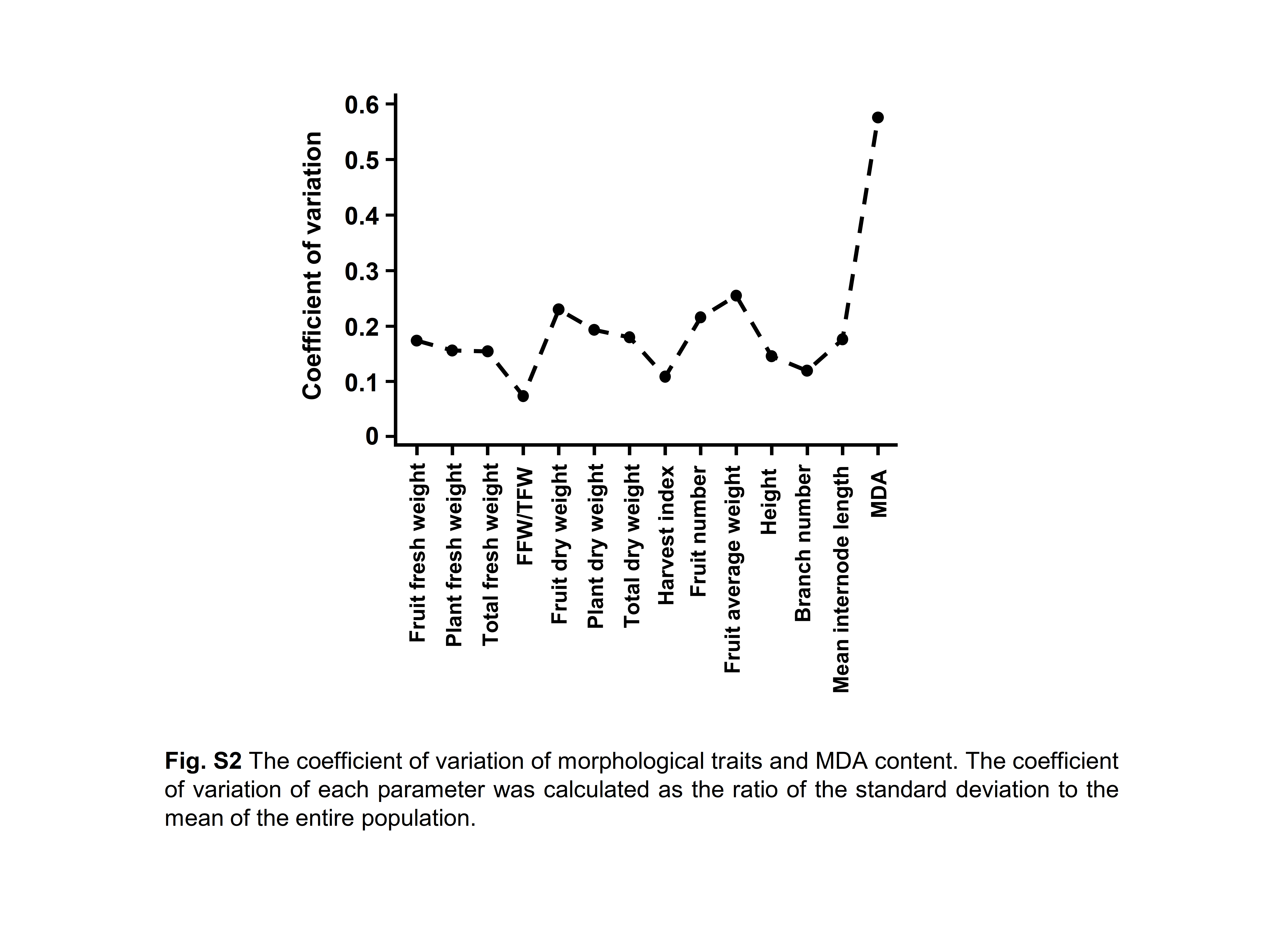

Supplement: Web_Material_uhac061 [file web_material_uhac061.zip › Fig. S2.tif]

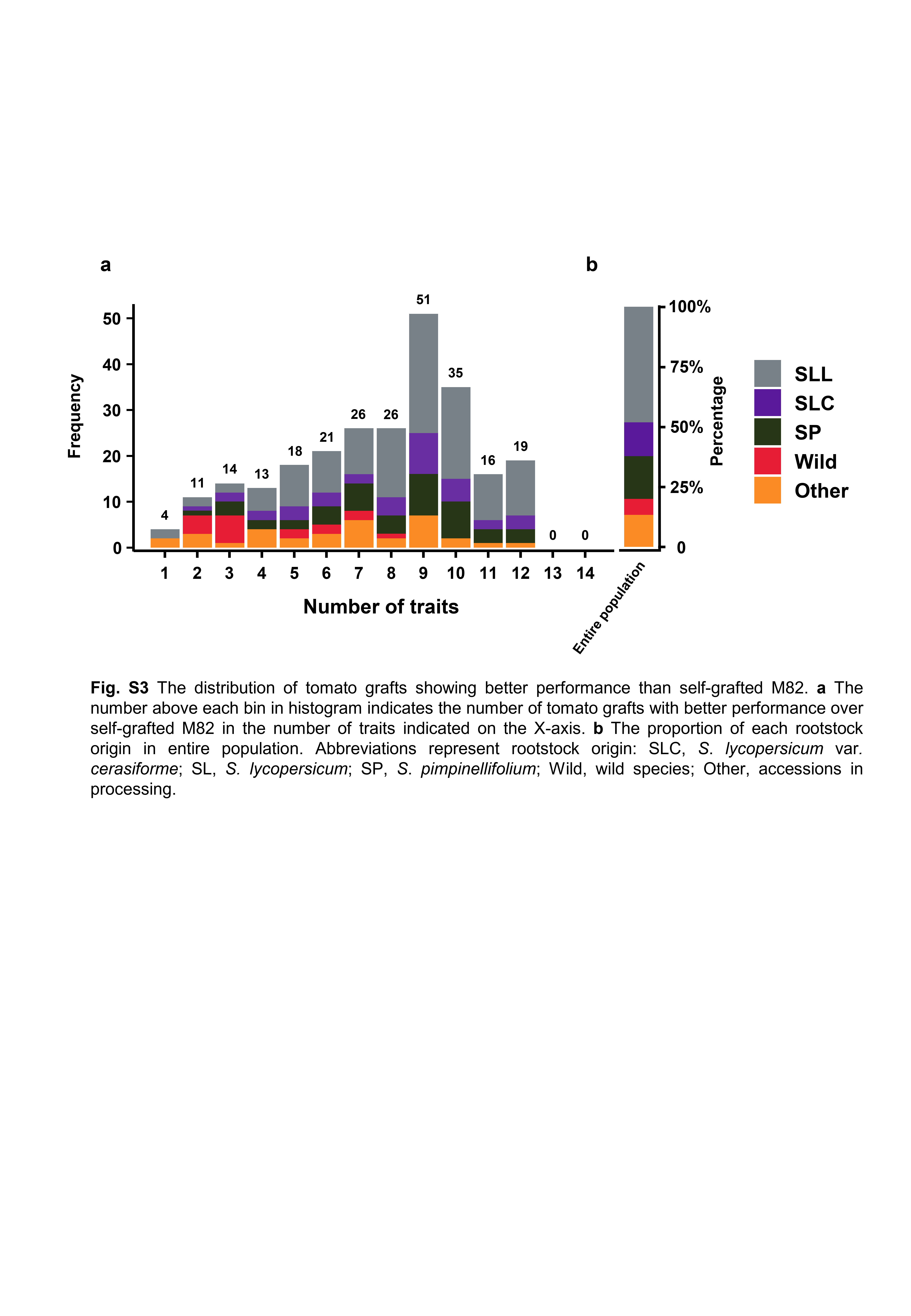

Supplement: Web_Material_uhac061 [file web_material_uhac061.zip › Fig. S3.tif]

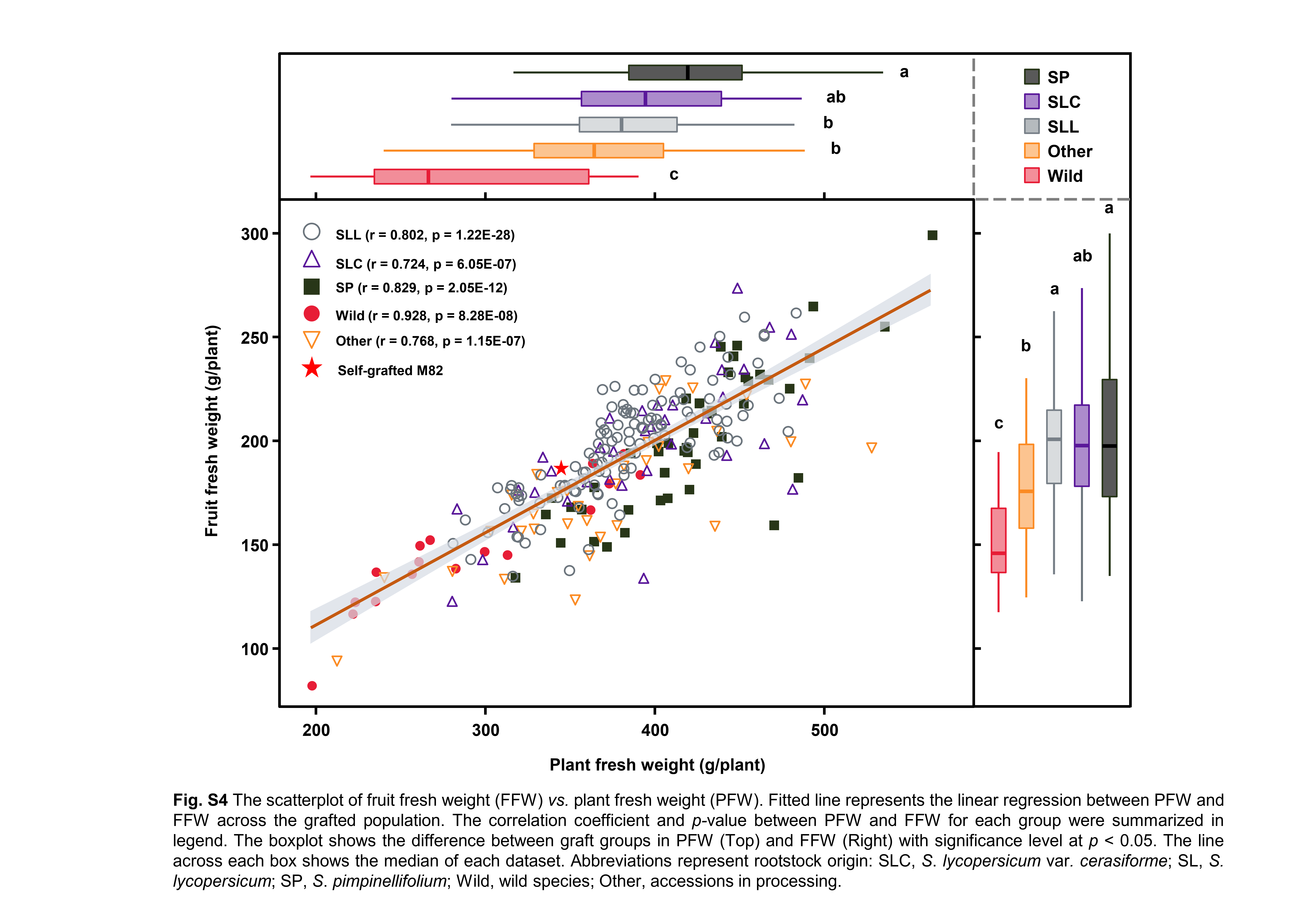

Supplement: Web_Material_uhac061 [file web_material_uhac061.zip › Fig. S4.tif]

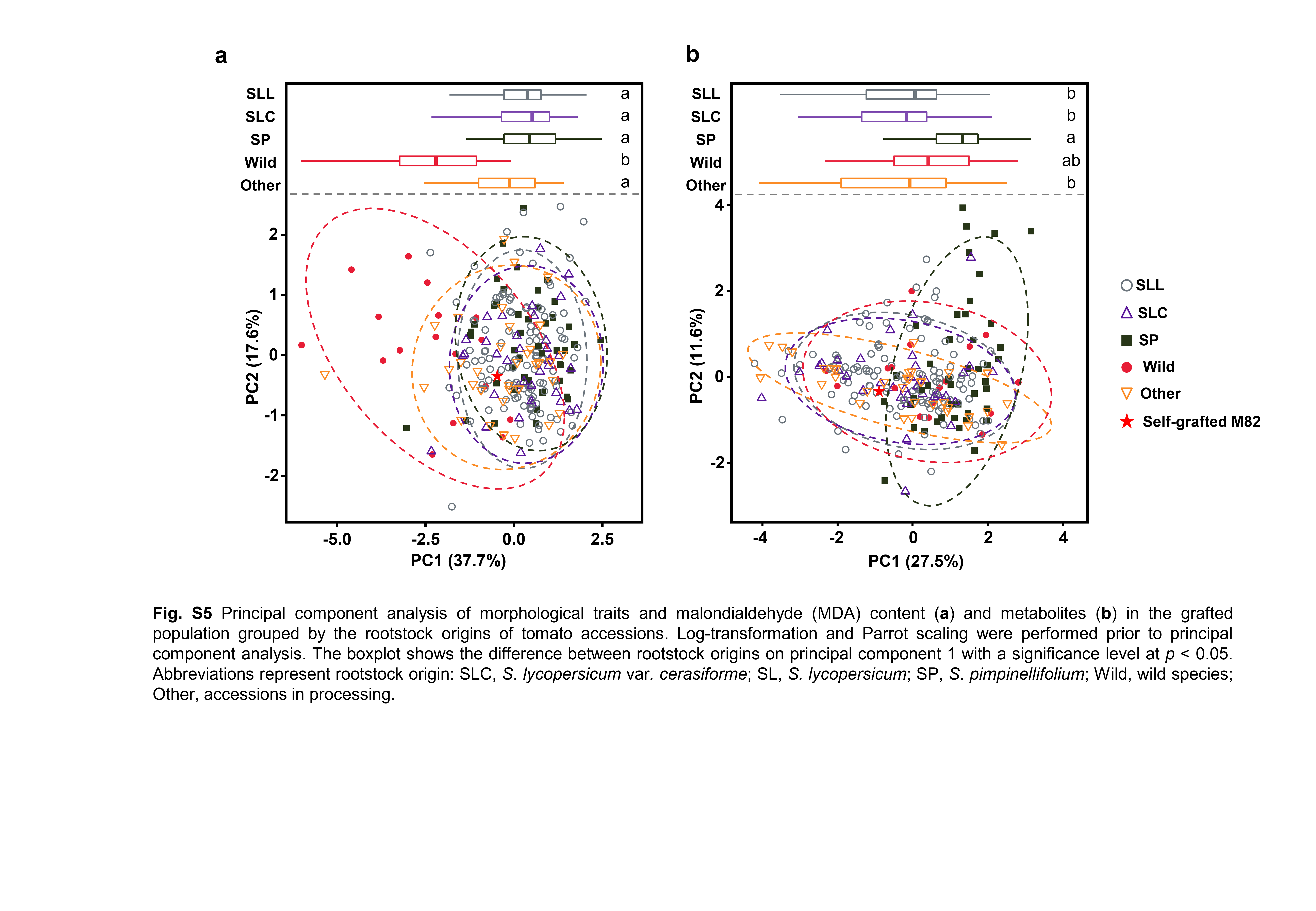

Supplement: Web_Material_uhac061 [file web_material_uhac061.zip › Fig. S5.tif]

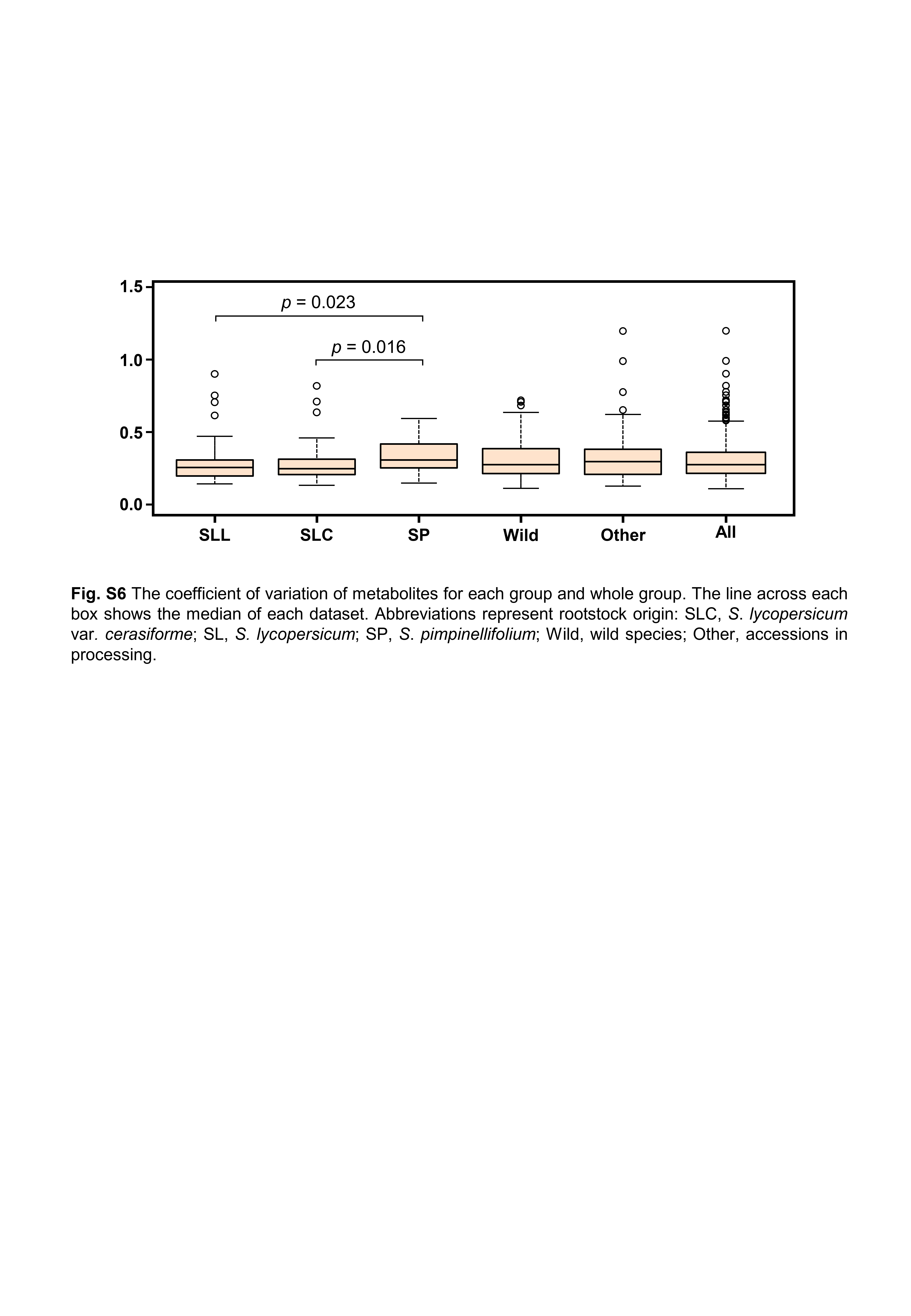

Supplement: Web_Material_uhac061 [file web_material_uhac061.zip › Fig. S6.tif]

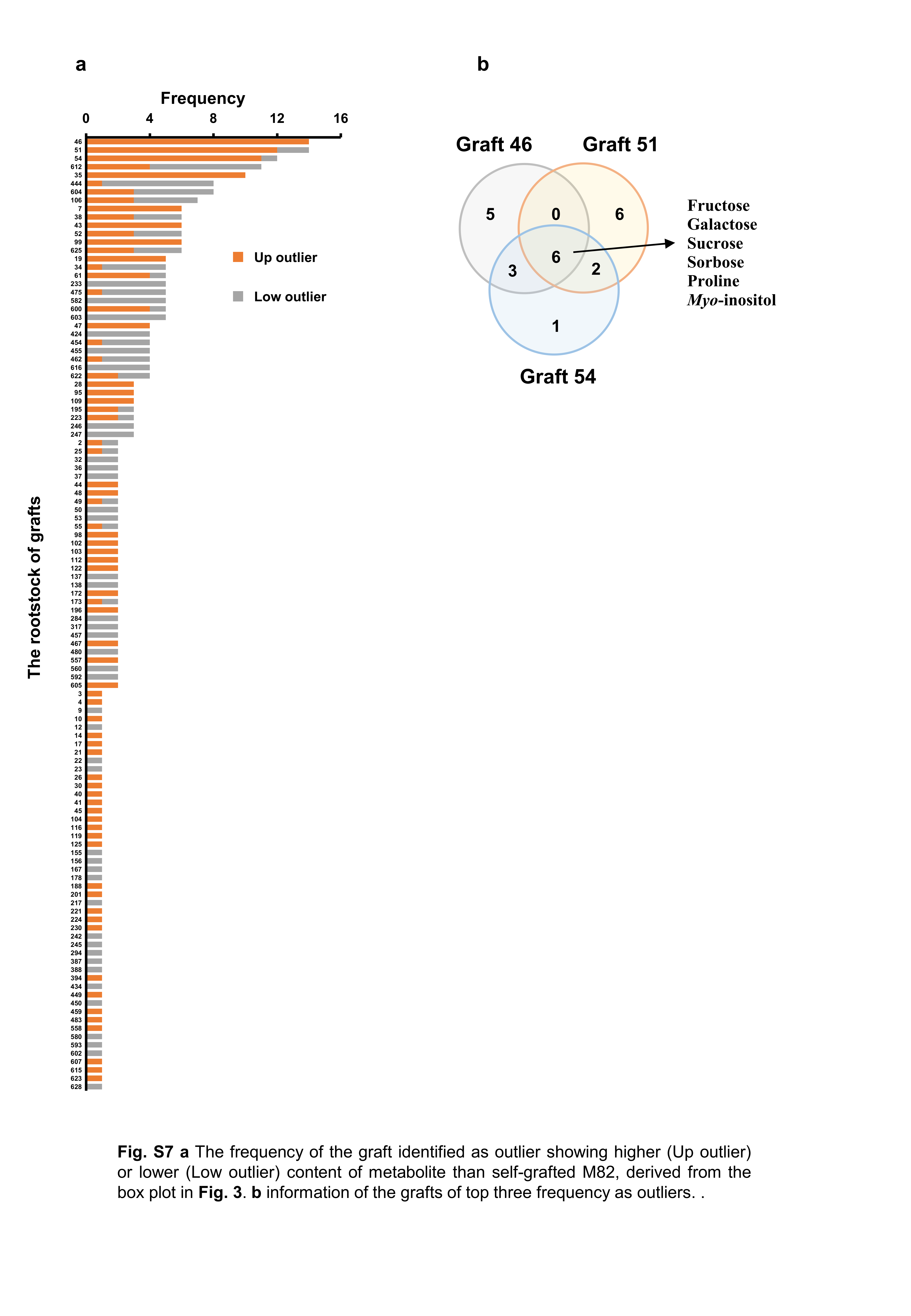

Supplement: Web_Material_uhac061 [file web_material_uhac061.zip › Fig. S7.tif]

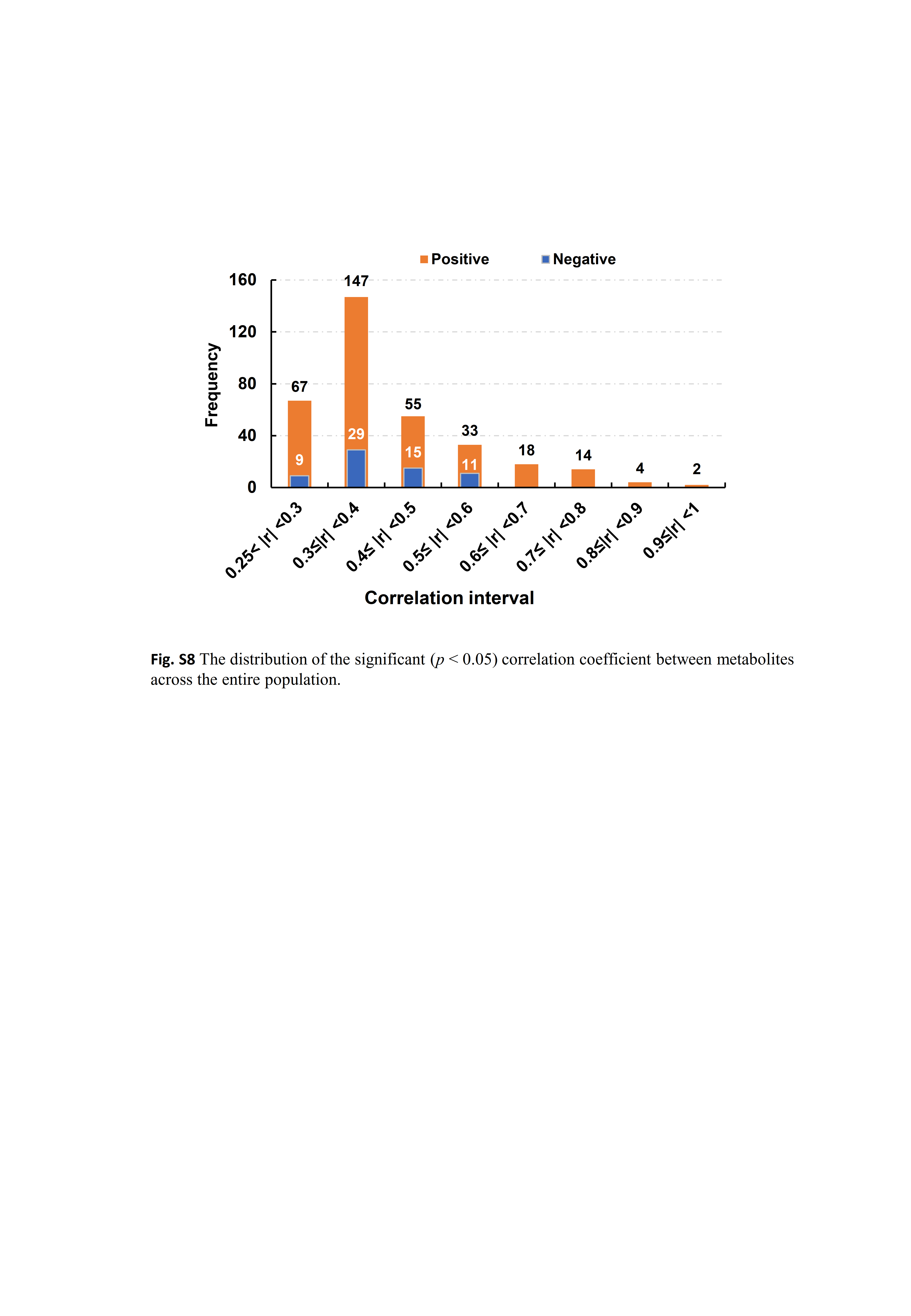

Supplement: Web_Material_uhac061 [file web_material_uhac061.zip › Fig. S8.tif]

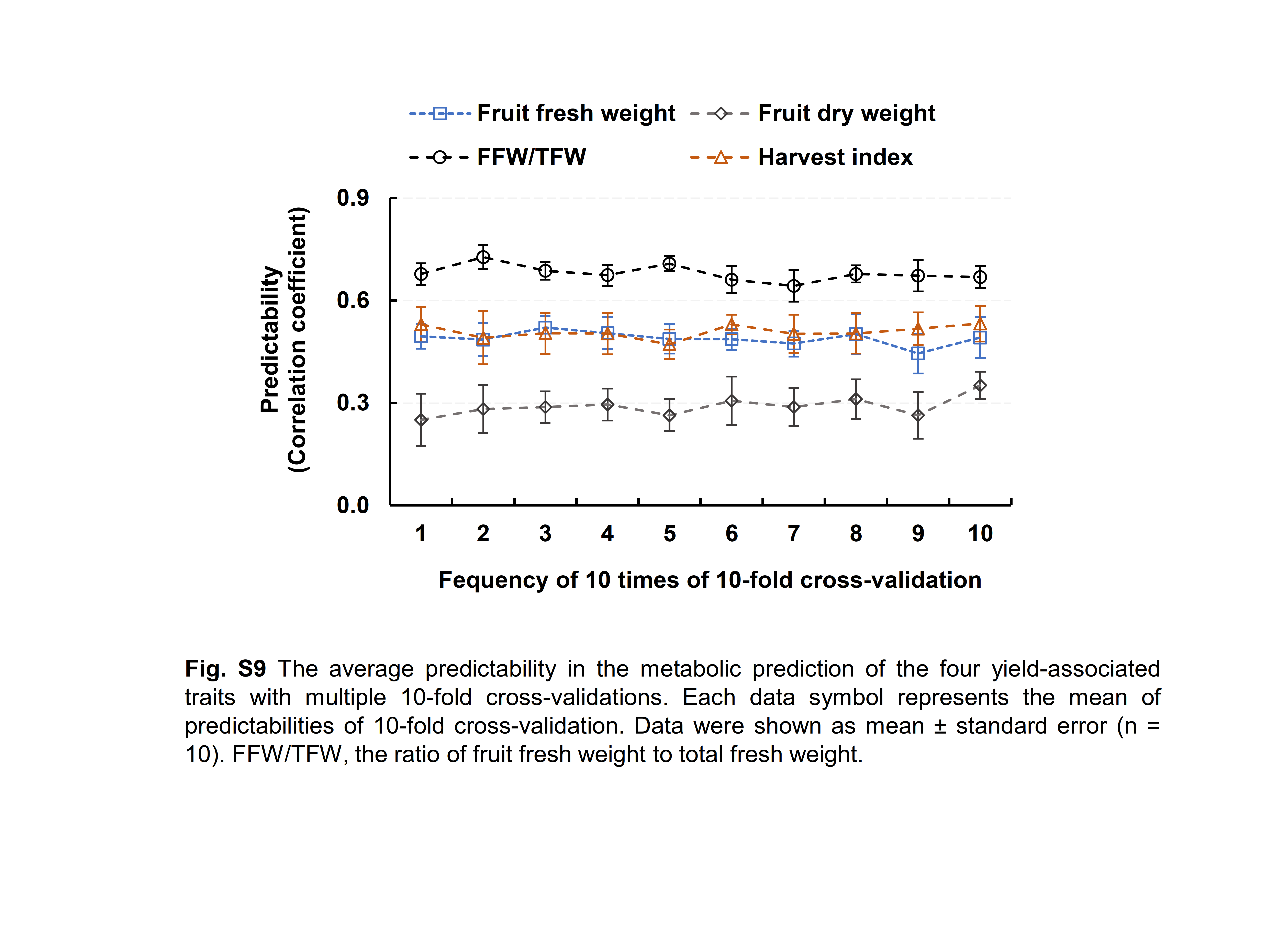

Supplement: Web_Material_uhac061 [file web_material_uhac061.zip › Fig. S9.tif]
